# Recent Progress in the Molecular Recognition and Therapeutic Importance of Interleukin-1 Receptor-Associated Kinase 4

**DOI:** 10.3390/molecules21111529

**Published:** 2016-11-13

**Authors:** Mahesh Chandra Patra, Sangdun Choi

**Affiliations:** Department of Molecular Science and Technology, Ajou University, Suwon 443-749, Korea; ml2mahesh@gmail.com

**Keywords:** IRAK4, TLR, inhibitor, X-ray crystallography, autoimmunity

## Abstract

Toll-like receptors (TLRs) are the most upstream pattern recognition receptors in the cell, which detect pathogen associated molecular patterns and initiate signal transduction, culminating in the transcription of pro-inflammatory cytokines and antiviral interferon. Interleukin-1 receptor-associated kinase 4 (IRAK4) is a key mediator in TLR (except for TLR3) and interleukin-1 receptor signaling pathways. The loss of kinase function of IRAK4 is associated with increased susceptibility to various pathogens, while its over-activation causes autoimmune diseases such as rheumatoid arthritis, systemic lupus erythematosus, and cancer. The therapeutic importance of this master kinase has been advocated by a number of recent preclinical studies, where potent inhibitors have been administered to improve various TLR-mediated pathologies. Increasing studies of X-ray crystallographic structures with bound inhibitors have improved our knowledge on the molecular recognition of ligands by IRAK4, which will be crucial for the development of new inhibitors with improved potencies. In this review, we briefly discuss the structural aspect of ligand recognition by IRAK4 and highlight its therapeutic importance in the context of TLR-associated unmet medical needs.

## 1. Introduction

**Figure 1:**
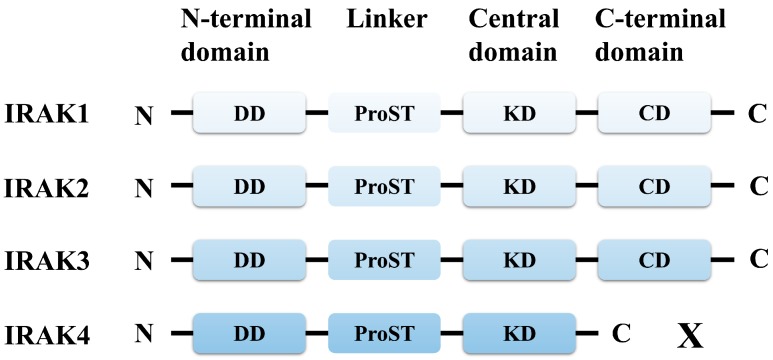
Domain architecture of interleukin-1 receptor-associated kinase (IRAK) family members. IRAK family members commonly contain an N-terminal death domain (DD) that interacts with myeloid differentiation primary response gene 88 (MyD88), a central kinase domain (KD) that phosphorylates IRAK1, and a C-terminal domain (CD), which is essential for TNF receptor associated factor 6 (TRAF6) recruitment. A proline, serine, threonine-rich (ProST) linker of unknown structure connects the DD and KD. ProST region is rich in proline, serine, and threonine residues that are heavily autophosphorylated, causing release of IRAK1-TRAF6 complex from the receptor complex. IRAK4 lacks a CD; thus, it is shorter than other IRAK members. IRAK2 and IRAK3 contain a pseudokinase domain that is kinase inactive.

Protein kinases are functionally diverse and undergo frequent mutations in the human genome. There are almost 518 genes that code for different protein kinases, which function in essential cellular pathways such as cell signaling, apoptosis, signal transduction, and propagation of immune responses [1]. Interleukin (IL)-1 receptor-associated kinase 4 (IRAK4) is a serine/threonine kinase that performs an important function of scaffolding and phosphorylation in toll-like receptor (TLR) and IL-1 receptor (IL-1R) signaling pathways [2]. TLR and IL-1R are two large families of pattern recognition and cytokine receptors, respectively, involved in inflammatory and immune signaling for the protection of host organism [3,4]. Detection of pathogenic patterns such as double stranded RNA (dsRNA), lipopolysaccharides (LPS), flagellin, and unmethylated CpG DNA, or cytokines such as IL-1β and IL-18, result in dimerization-driven signal transduction. TLR and IL-1R share a common toll/IL-1R (TIR) domain, which is crucial for recruiting the downstream adaptor molecule, myeloid differentiation primary response gene 88 (MyD88) [5]. Subsequently, MyD88 recruits interleukin-1 receptor-associated kinase (IRAK) family members through a complex assembly of death domains (DD) called ‘myddosome’ [6]. Four subtypes of IRAKs are known: IRAK1, IRAK2, IRAK3 (or IRAKM), and IRAK4. IRAK4 is the most upstream kinase of the IRAK subtypes, which recruits either IRAK1 or IRAK2 by trans-autophosphorylation of serine and threonine residues. IRAK1 or IRAK2, in turn, recruits TNF receptor associated factor 6 (TRAF6) and propagates signal transduction, leading to the activation of nuclear factor kappa-light-chain-enhancer of activated B cells (NF-κB) and mitogen activated protein (MAP) kinases [2,7]. IRAKM is specifically expressed in macrophages and monocytes and negatively regulates TLR signaling [8]. At the molecular level, IRAKM prevents dissociation of IRAK1 from MyD88 and formation of IRAK1-TRAF6 signaling complex. Recently, it was found that IRAKM can interact with the MyD88-IRAK4 complex, forming a myddosome superassembly that mediates TLR7-induced NF-κB activation [9]. Structurally, IRAK family members are composed of an N-terminal DD and a central kinase domain (KD), connected by a flexible linker of unknown structure, also known as proline, serine, threonine-rich (ProST) domain [10]. IRAK4 lacks a C-terminal domain, which is required by other three IRAKs for TRAF6 recruitment. It is believed that only IRAK4 and IRAK1 have intrinsic kinase activity, and the kinase activity of IRAK1 is dispensable for downstream adaptor recruitment. IRAK2 and IRAKM contain an inactive pseudokinase domain (Figure 1). Although sequence homology studies indicate that IRAKs are serine/threonine kinases, the phosphate ligand binding patterns and the structures of substrate binding sites are analogous to tyrosine kinase-like family members [1].

**Figure 2:**
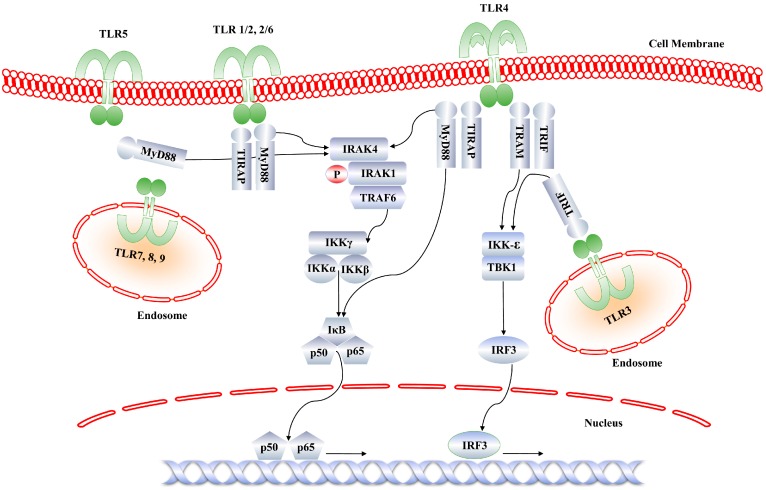
Toll-like receptor (TLR) signaling pathways. IRAK4) is the central kinase that mediates signal transduction from all TLRs, except TLR3 and TIR-domain-containing adapter-inducing interferon-β (TRIF)-dependent TLR4. MyD88 recruits IRAK4, which autophosphorylates and then phosphorylates IRAK1. The phosphorylated IRAK1 recruits TRAF6 and propagates signal transduction, resulting in the nuclear translocation of nuclear factor kappa-light-chain-enhancer of activated B cells (NF-κB).

Since IRAK4 functions in a MyD88-dependent pathway, TLR3 signaling requires a different set of adaptor and kinase molecules (Figure 2). Both MyD88 and IRAK4 bear a huge therapeutic importance in anti-inflammatory and anti-cancer drug development [11,12,13,14]. Genetic evidence has suggested that IRAK4 is a crucial factor in TLR/IL-1R signaling pathways. IRAK4 knockout mice have shown poor immune signaling and diminished response to IL-1 and TLR agonists. In humans, IRAK4 deficient phenotypes are highly susceptible to recurrent pyogenic bacterial infections. Because of IRAK4’s central role in propagating innate and adaptive immunity, IRAK4 inhibitors are considered valuable therapeutics in autoimmune and inflammatory disorders. Here, we review the recently reported intermolecular interactions between inhibitors and IRAK4. We highlight the key molecular determinants required for ideal ligand binding to IRAK4, based on published X-ray crystal structures. A brief overview of the therapeutic importance of IRAK4 has also been given. The information in this article might be useful for gaining an improved understanding of the structure and function of IRAK4.

## 2. Functional Role of IRAK4

The function of IRAK4 is reminiscent of the pelle protein of Drosophila, which mediates toll receptor signaling during embryonic development of the fly. Ligand recognition by TLR/IL-1R enables receptor dimerization and subsequent recruitment of the adaptor protein, MyD88, through TIR domain interactions. IRAK4 performs a dual role of scaffolding and phosphorylation through its N-terminal and C-terminal domains. The N-terminal DD associates with MyD88, while the C-terminal KD recruits IRAK1 through trans-autophosphorylation, which completes the ‘myddosome’ formation [6]. IRAK4 mutants with truncated N-terminal DD fail to associate with MyD88 and do not form the IL-1R signaling complex in response to IL-1 at the membrane, thus preventing recruitment of IRAK1 [15]. IRAK4 knock-out mice are completely resistant to LPS challenge and display a severe impairment of NF-κB mediated proinflammatory cytokine expression in response to TLR agonists or IL-1 [16]. IRAK4 kinase-dead knock-in mice demonstrate complete resistance to septic shock induced by TLR4 [17]. IRAK4 deficiency in humans is an autosomal recessive disorder, which makes patients susceptible to infections caused by Gram-positive pyogenic bacteria such as *Streptococcus pneumoniae* [18].

The kinase activity of IRAK4 is essentially required for LPS and CpG oligodeoxynucleotide-induced innate immune signaling [17,19], resulting in the activation of NFκB and MAP kinases. However, studies have shown that early activation of NF-κB is not affected by kinase inactive IRAK4 [17]. Since IRAK4 exclusively functions in the MyD88 signaling pathway, TLR4-mediated interferon response factor 3 (IRF3) activation is unaltered. This indicates that IRAK4 function does not affect interferon production, which is the end product of TLR3 and TRIF-dependent TLR4 pathways. It has also been demonstrated that LPS stimulation does not impact a particular set of genes, but it certainly reduces the expression of pro-inflammatory genes, including tumor necrosis factor-α (TNF-α) [17]. Malfunction of TLR and/or IL-1 receptor signaling causes systemic lupus erythematosus (SLE), rheumatoid arthritis (RA), psoriasis, gout, and inflammatory bowel disease [20,21,22,23].

IRAK4 is activated by autophosphorylation of Thr342, Thr345, and Ser346 residues within the activation loop [7]. Recently, Thr352 has also been identified as an additional phosphorylation site [24]. Stimulation with TLR/IL-1R agonists (such as LPS and resiquimod (R848)) enables recruitment, dimerization, and phosphorylation of IRAK4, indicating a role of IRAK4-DD in MyD88 attachment, which provides a platform for activation of the kinase function of IRAK4 [25]. Surprisingly, inhibition of IRAK4 using a dual IRAK1/4 inhibitor blocked IRAK4 auto-phosphorylation, but activation of NF-κB and MAP kinase remained unaltered in fibroblasts and monocytes. However, IRAK4-knockout cells failed to activate NF-κB and MAP kinases in response to agonists, such as IL-1β. Furthermore, a cell-type specific effect of IRAK4 activation has been observed: pharmacological inhibition of IRAK4 failed to produce cytokines in primary monocytes, while dermal fibroblasts remained unaffected [24].

## 3. Structure of Kinase Domain of IRAK4

**Figure 3:**
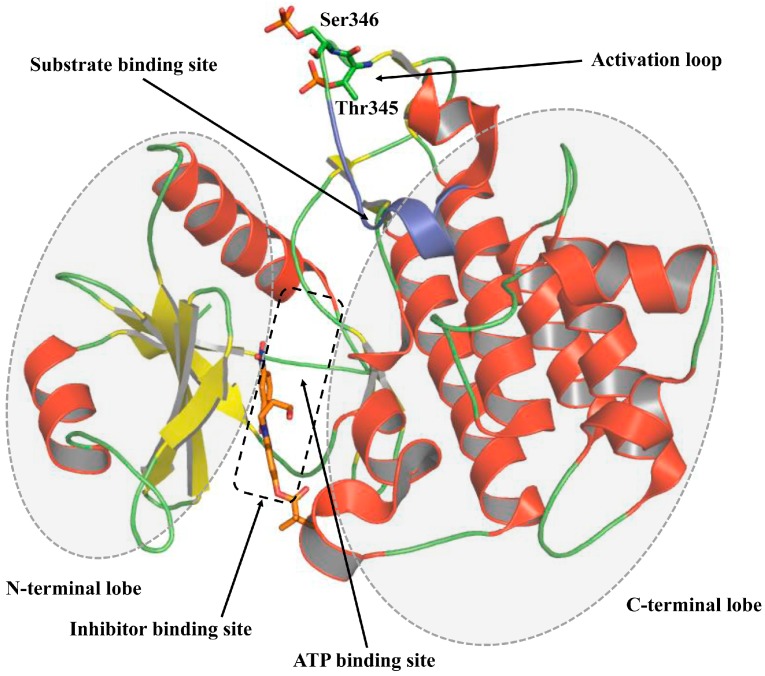
Overall structure of IRAK4 kinase domain. IRAK4 kinase domain consists of an N-terminal and a C-terminal lobe with the ATP-binding pocket lying between the two lobes. The binding site of IRAK4 inhibitors overlaps with that of ATP. The region highlighted in blue color mediates substrate (IRAK1) binding and phosphorylation.

The X-ray crystal structure of human IRAK4 DD has been reported [26]. The first crystal structure of IRAK4 KD was concurrently reported by Wang et al. [27] and Kuglstatter et al. [28]. The structure revealed four distinct kinase specific structural regions: activation loop, substrate binding site, ATP binding site, and inhibitor binding region (Figure 3). The IRAK4 KD consists of an N-terminal lobe with five antiparallel β-sheets and an α-helical C-terminal lobe. The ATP binding pocket is found between the two lobes, similar to the observations in other protein kinases. IRAK4 lacks a C-terminal extension, which is required by other IRAK members for TRAF6 interaction. Although IRAKs have identical three dimensional folds, the sequence similarity among them ranges between 31% and 32%. However, the ATP binding pocket residues show a higher sequence homology: IRAK4 shares the highest sequence identity (at 93%) with IRAK1, indicating a common ATP binding pattern. The N-terminal lobe has a tyrosine residue (Tyr262) that acts as a gatekeeper, preventing access to the hydrophobic pocket formed by Asp-Phe-Gly (DFG) motif and helix α C at the back of ATP. Another interesting feature of this lobe is the presence of an N-terminal extension of unknown function, commonly known as the Schellman loop [29]. The gatekeeper tyrosine (Tyr262) is exclusive to the IRAK family, and this residue has been exploited in structure based drug design for kinase selectivity of IRAK4. The hydroxyl group of its bulky side chain forms a hydrogen bond (H-bond) with Glu233 located on helix α C, thus preventing ligand access to the hydrophobic back pocket. Moreover, the interaction between Tyr262 and Glu233 holds the KD in active DFG-in conformation.

## 4. Structure of IRAK4 with Bound Inhibitors

**Figure 4:**
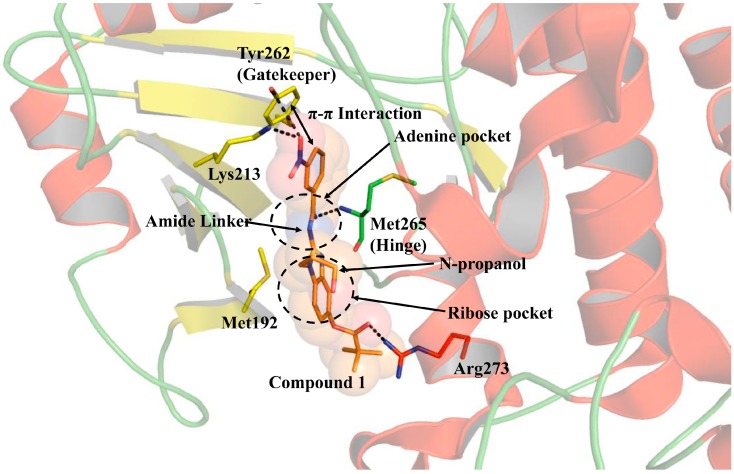
Interaction of compound 1 (2NRU) with the catalytic site of IRAK4 kinase domain. Compound **1** (*N*-acyl 2-aminobenzimidazole) displays three kinase-specific interactions: a π-π interaction with the gatekeeper Tyr262, an H-bond with the backbone of hinge Met265, and an H-bond with the catalytic Lys213. Compound **1** perfectly occupies the ATP binding site with the amide linker positioned at the ATP-adenine pocket and the N-propanol substitution positioned at the ATP-ribose binding area.

Several crystal structures of IRAK4 have been reported with or without bound ATP competitive inhibitors. These crystal structures provide information regarding the general pharmacophore within the ATP binding pocket, as well as the chemical groups of the ligand that are complementary to the active site residues. The first crystal structure of IRAK4 has been co-crystallized with a potent inhibitor compound **1** (*N*-acyl 2-aminobenzimidazoles), identified through high-throughput screening (HTS), followed by structure activity relationship (SAR) study [30]. Compound **1** has a structure of 3-nitro phenyl moiety, connected by an amide linker to a benzamidazole scaffold (Figure 4). The inhibitor binds IRAK4 in an extended conformation with the carbonyl of the amide linker forming an H-bond with the backbone amide of the hinge Met265. The phenyl ring forms an aromatic stacking (π-π) interaction with the gatekeeper Tyr262. The catalytic Lys213 and Tyr262 residues form one H-bond each with the nitro group of the inhibitor. The amide linker of the ligand occupies the adenine binding site, while the N-propanol replacement is positioned toward the ribose binding site. The benzimidazole ring makes a van der Waals contact with Met192 of the phosphate binding loop (P-loop). SAR studies around the amide linker and benzimidazole moieties revealed their importance in potent IRAK4 inhibition, as modifications at these regions drastically reduced the inhibitory activity of the compound [31]. The trimethyl acetyl ester substitution is solvent-exposed and forms an H-bond with Arg273 from helix αD. Among all these interactions, the kinase-specific interactions are the H-bonds at the hinge, π-stacking with the gatekeeper, and the H-bond interaction with the catalytic loop.

**Figure 5:**
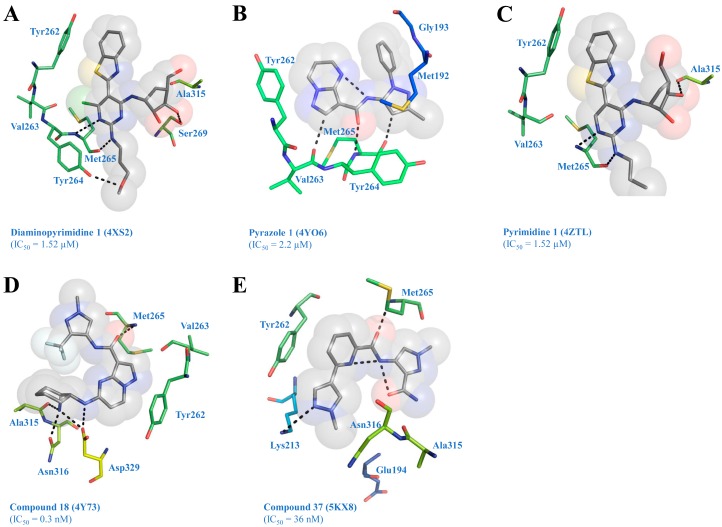
Interactions of co-crystallized inhibitors with IRAK4 residues. The figure describes the typical interactions that the co-crystallized ligands (**A**) diaminopyrimidine 1, (**B**) pyrazole 1, (**C**) pyrimidine 1, (**D**) compound 18, and (**E**) compound 37 make with the catalytic site of IRAK4. The most unusual interaction was observed between the lead compound pyrazole **1** and IRAK4. Pyrazole **1** forms three H-bonds with the hinge backbone region, in contrast with other inhibitors, which form one. All inhibitors bind IRAK4 in a nearly planar orientation, which is a crucial feature required for π-stacking with the gatekeeper Tyr262. IC_50_ = inhibitory concentration.

McElroy et al. [32] have identified a novel diaminopyrimidine series of inhibitors using HTS. X-ray crystallography of the preliminary hit was performed to serve as a guide in further SAR studies. The inhibitor competitively binds to the ATP pocket of IRAK4. The pyrimidine C2 amino group and N3 nitrogen form critical H-bonds with amino and carbonyl groups of hinge Met265, respectively (PDB ID: 4XS2). The pyrimidine C4 chlorine makes a weak polar contact with the carbonyl group of Val263. An intramolecular H-bond occurs between the C6 amino group of pyrimidine and nitrogen atom of benzothiazole, which maintains the benzothiazole in a planar conformation. This allows a π-π interaction with the phenol ring of gatekeeper Tyr262 (Figure 5A). The hydroxyl groups at C30 and C50 positions of carboribose form H-bonds with the hydroxyl and carbonyl groups of Ser269 and Ala315, respectively. The hydroxyl group of Tyr264 was observed to engage in an H-bond interaction with the oxygen atom of the methoxypropyl side chain. Further, McElroy and colleagues [33] have designed a series of pyrazoles containing potent, selective, and orally bioavailable IRAK4 inhibitors, using an HTS campaign. X-ray crystallography revealed an interesting binding mode of pyrazole **1** complexed with IRAK4 (PDB ID: 4YO6). Pyrazole 1 forms three H-bonds with the hinge Met265 and an intramolecular H-bond between the amide linker and nitrogen of pyrimidine, maintaining a planar conformation of the inhibitor (Figure 5B). The pyrazolopyrimidine ring interacts with the gatekeeper Tyr262 through a partial π-π interaction. The pyrazole phenyl substituent is located in front of the peptide bond between Met192 and Gly193 near the P-loop (glycine rich loop). The C3 substituent on the pyrazole ring faces the solvent accessible area, which was later subjected to SAR studies and exhibited improved IRAK4 activity.

Seganish et al. [34] have identified aminopyrimidin-4-one series of IRAK4 inhibitors using HTS, followed by structure enabled design. X-ray crystallography has revealed a novel binding mode of this series (PDB ID: 4ZTL). The C2 position of the inhibitor has been explored through SAR analysis, and the carboribose replacement has generated improved ligands with excellent kinase selectivity and good oral bioavailability in rats. In the X-ray co-crystal structure of pyrimidine **1**, there is a small pocket close to C6 position and Val263 backbone carbonyl (Figure 5C). This position was replaced by a hydroxyl group that showed improved interaction with Val263 carbonyl group through an H-bond.

Lim et al. [35] have developed 5-amino-*N*-(1*H*-pyrazol-4-yl)pyrazolo[1,5-*a*]pyrimidine-3-carboxamide inhibitors using HTS, followed by SAR analysis on fifth position of pyrazolopyrimidine ring and third position of the pyrazole ring. Sequential modifications generated a lead molecule, *N*-(3-carbamoyl-1-methyl-1*H*pyrazol-4-yl)pyrazolo[1,5-*a*]pyrimidine-3-carboxamide, which displayed excellent drug-like properties, potency, kinase selectivity, and good oral bioavailability. In the crystal structure (PDB ID: 4Y73), the inhibitor (compound **18**) forms an H-bond with the side chain oxygen of Asn316 through its 2*S*-amino group attached to the cyclohexane moiety. The 1R-amino group is involved in an H-bond with the side chain carboxyl group of Asp329. The pyrazolopyrimidine core interacts at an angle with the phenol side chain of the gatekeeper Tyr262 residue (Figure 5D). There is a single polar hinge interaction provided by the amide oxygen with the backbone NH of Met265. The trifluoromethyl (CF_3_) group stacks intramolecularly with the cyclohexyl ring on the other side of the compound.

Hanisak et al. [36] have identified and performed SAR studies on a novel series of pyrazole-based inhibitors with potent IRAK4 activities and good ligand efficiencies. This series displayed low off-target activity and desirable rat clearance data. The optimization of the molecules in this series were extended from a previously identified pyrazole containing ligand class [33]. In the crystal structure, the ligand (compound **37**) shows both intramolecular and intermolecular H-bonds (PDB ID: 5KX8). The amine of Met265 from the hinge region forms an H-bond with the carbonyl of ligand (Figure 5E). This interaction has been shown to influence the kinase selectivity of the ligand.

## 5. Development of IRAK4 Inhibitor (Non-Crystallographic Study)

Although IRAK4 is an important and interesting therapeutic target to treat inflammatory and autoimmune diseases, identification and development of selective inhibitors is challenging. The highly conserved structure of IRAK4 catalytic domain itself affects ligand selectivity. In addition, several groups found it difficult to obtain small molecules with properties suitable for animal trials [27,37]. Earlier, Buckley et al. [38,39,40], in three subsequent publications, reported a series of thiazole amide, 2-aminopyrimidine, and imidazo[1,2-*a*]pyridino-pyridine IRAK4 inhibitors using HTS, followed by SAR studies. These classes of inhibitors were initially designed for a c-Jun N-terminal kinase (JNK) kinase program, which inhibited IRAK4 as an off-target effect. Computational analysis using a homology model of IRAK4 revealed key intermolecular interactions between the ligand and the ATP binding pocket of the protein. A thiazole amide was identified from screening a library of small molecules against IRAK4, as an initial hit with IRAK4 inhibitory concentration (IC_50_) = 2.8 μM [40]. Computational analysis using docking and homology modeling showed that the amide linker forms an H-bond with the hinge Met265. The terminal pyridine shows a π-stacking interaction with the gatekeeper Tyr262. The aniline ring was subjected to SAR analysis, resulting in a 2-methoxy-4-piperidin-1-yl aniline substituent (compound **4a**) that showed suitable properties for an *in vivo* study. Furthermore, in a JNK kinase program, 2-aminopyrimidine-based inhibitors showed significant potency towards IRAK4 as an off-target effect [38]. Further development of this series resulted in two highly selective molecules: imidazo[1,2-*a*]pyridino-pyridine and benzimidazolopyridine [39]. Computational docking onto an IRAK4 homology model showed the imidazolepyridine nitrogen (N1) bound to the hinge Met265. Compounds **5** and **6** from this series were proven to be useful IRAK4 inhibitors, using SAR analysis.

Kelly et al. [41] have discovered and performed *in vivo* pharmacological characterization of potent, highly selective, and bioavailable thienopyrimidine class of IRAK4 inhibitors to treat autoimmune diseases and B cell malignancies. Initial virtual screening of a commercially available chemical library containing 1.3 million compounds suggested a series of thienopyrimidine compounds as potential hits. Two compounds, namely ND-2110 and ND-2158, were confirmed to inhibit IRAK4, based on a kinase selectivity test against 334 kinases. The compounds bind competitively to the ATP binding pocket, show good solubility, cell permeability, and pharmacokinetic profile. ND-2158 and ND-2110 inhibited IRAK4 and NF-κB activity in activated B cell–like (ABC) subtype of diffuse large B cell lymphoma (DLBCL) cell lines with L265P mutation in MyD88. The compounds blocked LPS and CpG induced TNF-α production, as well as ameliorated collagen-induced arthritis, and gout formation in Xenograft DLBCL mice. The authors also studied a combined application of ND-2158 and a B cell signaling inhibitor, ibrutinib, showing synergistic effect on mice tumor growth. This treatment option will benefit patients suffering from B cell lymphomas having L265P mutation or autoimmune diseases. Intriguingly, why the IRAK4 inhibitors were only effective on ABC-DLBCL cell lines with L265P mutation needs to be investigated [42].

Dou et al. [43] have designed a new benzenediamine derivative, named FC-99, which was found effective in experimental sepsis by inhibiting LPS-mediated macrophage activation. The authors have screened a library of natural products and suggested that FC-99 directly targets IRAK4 by performing a number of computational and experimental analyses, including microarray and computer simulations. FC-99 rescued mice from lethal sepsis and showed promising results on experimental sepsis, indicating that it might be a potent therapeutic for the treatment of sepsis and other inflammatory disorders.

Tumey et al. [44] found a lead 9-cyano-indolo[2,3-*c*]quinolone (compound **4**) from an HTS, showing an IRAK4 potency of IC_50_ = 7.4 nM. However, this exceptional potency was questionable because of trace contamination of the sample by isomeric cyano-indolo[2,3-*c*]quinolines (compound **4**/**5**). A computational docking study of compound **4** on IRAK4 catalytic domain revealed that the quinoline nitrogen forms an H-bond with the ‘hinge’ Met265, similar to other related kinase inhibitors (i.e. quinoline and quinazoline). The nitrile attached to the cyanophenyl ‘headpiece’ forms an H-bond with the catalytic Lys213. The indolo[2,3-*c*]quinoline shows a partial π-stacking interaction with the unique IRAK-specific gatekeeper Tyr262. The dimethoxy segment of the inhibitor is exposed to solvent accessible region of the enzyme. After several rounds of SAR analysis, two compounds, named **26** and **32**, were obtained, which showed excellent pharmaceutical profiles, increased solubility, and whole blood potency. These inhibitors were shown to inhibit LPS-induced TNF-α production in a murine model.

## 6. Selective Kinase Inhibitor Development Targeting Inactive or Allosteric Sites of IRAK4

To date, inhibitors of the inactive state of IRAK4 have not been designed, probably because of the inherent difficulty in obtaining crystal structure of DFG-out kinases. The activation loop in the DFG-out conformation is highly dynamic and may exist in more than one conformation. This may lead to poor kinase selectivity through structure-based design. A large number of crystal structures need to be solved in the inactive state to understand the possible conformations of the activation loop, in particular, the orientations of Phe and Asp residues. However, a recent study has suggested that the inactive state conformation of kinases can be predicted computationally using the active state conformation of activation loop [45]. A slight modification of the activation loop, and a 5–15° manual rotation of the N-terminal domain, could result in an inactive state conformation of the kinase domain, which could be virtual screened for determining potential inhibitors.

**Figure 6:**
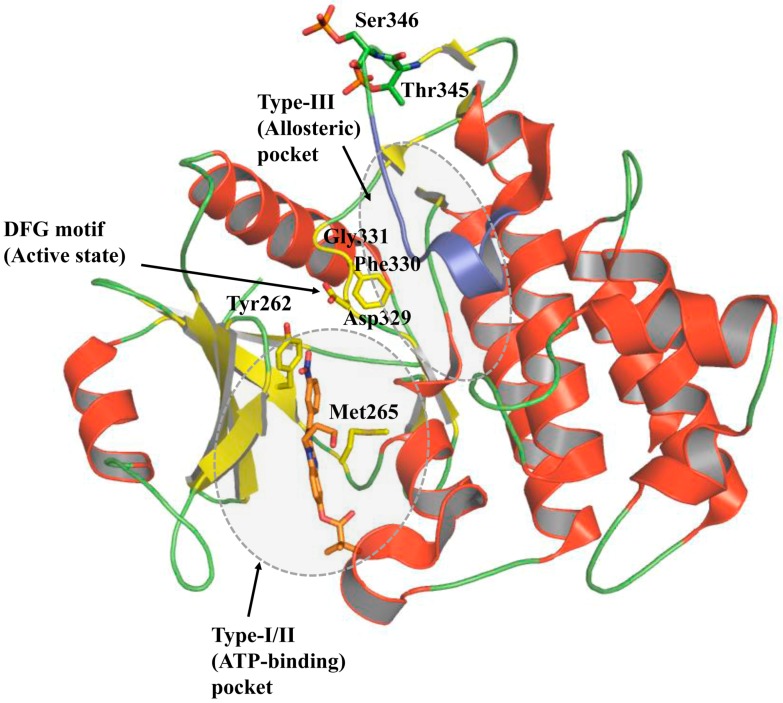
Illustration of Type-I, Type-II, and Type-III binding pockets of IRAK4 kinase domain. Type-I and type-II binding pockets are determined by the positions of Phe and Asp residues from the Asp-Phe-Gly (DFG) motif. Type-I binding site is formed in DFG-in condition, where the side chain of Phe faces towards the catalytic center, while the side chain of Asp faces in the opposite direction. Type-II binding site is formed when Phe and Asp side chains rotate 180°. Type-III binding site is the allosteric site, where IRAK1 is phosphorylated by IRAK4.

Currently, the protein data bank (PDB) houses more than 200 protein kinase domain structures, including a number of co-crystallized inhibitors, indicating the increased interest in elucidation of crucial protein domains that are important for drug designing. All these structures have highlighted three major states that inhibitors can target: (1) ATP-competitive active (type I) or the inactive (type II) state; (2) allosteric state (type III); and (3) surface pockets that interact with kinase regulators (type IV) (Figure 6). A number of design strategies could be employed for developing IRAK4 selective inhibitors through a more rational approach. The ATP-binding site has been the focus of IRAK4 drug discovery. This site is highly conserved and structurally rigid, which is necessary for catalytic activity. In the catalytically active state, also known as DFG-in state, the side chain of Phe faces toward the catalytic site, while that of Asp points outward. This orientation of Phe and Asp gives the catalytic core a proper shape for efficient phosphorylation. Targeting the catalytic site with ATP competitive inhibitors has been a challenging task. In contrast, the kinase inactive state, in which the side chains of Phe and Asp flip about 180°, is structurally diverse and flexible. This state has been successfully explored to discover selective ligands for several kinases [46]. However, IRAK4 is yet to be proven to exist in an inactive state. The second kind of inhibitors are termed type II, which bind to the inactive state of a kinase, where the DFG motif in folded into an inactive state. In this state, the inhibitors bind to the ATP pocket and keep the kinase in an inactive state [47]. Type III inhibitors bind the kinases at allosteric sites, and they displace the ATP out of the catalytic site without competing for ATP. In case of IRAK4, this site could be the substrate binding site where IRAK1 attaches. Type IV inhibitors target the surface, where other adaptors or regulators bind, and perform signal transduction. The MyD88 binding surfaces on the DD and KD of IRAK4 can be utilized to build small, peptide based inhibitors. Recently, decoy peptides derived from TIR domains of TLR and adaptor proteins have been proven to be efficient immune signaling blockers [48,49,50]. The inhibitors targeting type III and type IV binding sites could be more specific and selective, as these sites are usually unique to a given kinase [51]. Interestingly, a recent study has found that type II inhibitors may be less selective than type I inhibitors, because of multiple conformations of the activation loop in the inactive state of the kinase domain [47].

## 7. Therapeutic Importance of IRAK4

Innate immune receptors are the frontline of defense against pathogenic invasion. Targeting these receptors has been of primary interest in drug designing. The end result of innate immune signaling pathway is the production of pro-inflammatory cytokines, such as TNF-α, IL-6, and interferons, in response to bacterial and viral molecular patterns. Toll-like receptors, the most widely studied pattern recognition receptors of innate immune system, recognize pathogen associated molecular patterns, as well as self-generated molecular patterns, such as nucleic acids (DNA/RNA), proteins (e.g., high mobility group box 1 HMGB1), and advanced glycated end-products (AGE) [52]. The serine/threonine kinase IRAK4 is a ubiquitously expressed enzyme, involved in the regulation of immune signaling initiating from TLR and IL-1R receptor families. Kinase-dead knock-ins and IRAK4 knockout mice showed reduced expressions of TLR and IL-1R induced pro-inflammatory cytokines [19,53,54]. Mice with kinase-inactive knock-in IRAK4 are completely resistant to antigen-induced arthritis (AIA) and serum transfer-induced (K/BxN) arthritis [55]. Similarly, IRAK4 deficient humans are unresponsive to TLR and IL-1R ligands [56]. However, these patients are susceptible to gram-negative bacteria, virus, and fungal infections. Sensitivity to gram-positive infections is recovered as the patient advances toward adolescence, implying alternate innate immune mechanisms do exist in the absence of IRAK4 [57]. These findings indicate that blockade of IRAK4 kinase function has a therapeutic value without affecting the normal immune response of the cell. Additional data point toward a possible application of IRAK4 inhibitors as anti-atherosclerotic agents [58].

Recently, it has been shown that IRAK4 is involved in the activation of NLRP3 inflammasome in macrophages of mice exposed to Listeria monocytogenes [59,60]. The sequential binding of IRAK4 and IRAK1 to TLR4/6 heterodimer causes activation of NLRP3 inflammasome, while deletion of IRAK4 or IRAK1 fails to activate NLRP3 inflammasome. Kang et al. [61] found that treatment with drugs—cinnamaldehyde and allopurinol—inhibited NLRP3 inflammasome activation in the pathogenesis of fructose-induced cardiac injury. These results suggest that inhibition of TLR4/6-IRAK1/4 signaling using IRAK4 or IRAK1/4 dual blockers may reduce NLRP3 inflammasome activation in fructose-induced myocardial cell inflammation.

Dysregulation of TLR signaling mediated by IRAK family members has been hypothesized as a vital factor in the initiation and development of cancer. A number of efforts have been made to develop IRAK4 and IRAK1 inhibitors aimed at cancer treatment [62,63]. Constitutive phosphorylated states of IRAK4 and IRAK1 have been reported in melanoma patients [64]. A combined treatment of vinblastine and small interfering RNAs (siRNAs) inhibited IRAK4 and IRAK1, showing reduced tumor growth and increased survival rate of melanoma xenograft model of mice. Therefore, a combinatorial approach, targeting IRAK4 and IRAK1, would be a promising endeavor to reduce tumor progression in patients. Evidence of drug conjugates containing a dual IRAK1/4 kinase inhibitor, combined with a Bruton’s tyrosine kinase (BTK) inhibitor resulted in the inhibition of NF-κB [65]. To this end, increasing data strongly indicate that IRAK inhibitors could be a potential treatment for the NF-κB dependent B cell lymphoproliferative disorder called Waldenstrom’s macroglobulinemia [62]. IRAK4 is also implicated in the regulation of vascular diseases. Genetic evidence suggests that loss of function of IRAK4 inhibits the formation of vascular lesions in double knockout apolipoprotein E (apoE-/-) mice [58]. LPS stimulation activates IRAK4 in vascular smooth muscle cells (VSMC), resulting in increased proliferation, migration, and secretion of monocyte chemoattractant protein 1 (MCP-1), an inflammatory cytokine. Knocking down of IRAK1/4 with either siRNA or inhibitors causes downregulation of NF-kB activity and decreased MCP-1 expression [66].

IRAK1/4 signaling has been associated with neointimal formation after carotid balloon injury. Bai et al. [67] has shown that *in vivo* attenuation of neointimal formation and fibrotic remodeling can be achieved using an IRAK1/4 dual inhibitor, *N*-(2-Morpholinylethyl)-2-(3-nitrobenzoylamido)-benzimidazole. The inhibition of IRAK1/4 was found to suppress production of TNF-α, IL-1β, and migration and proliferation of VSMC in injured arteries *in vitro*. Elevated levels of IRAK1 and IRAK4 mRNA have been found in T acute lymphoblastic leukemia (T-ALL) cells. Inhibition of IRAK4 and IRAK1, either with short hairpin RNA (shRNA) or with an IRAK1/4dual inhibitor, resulted in decreased expression of IL-17 and interferon-γ (IFN-γ), as well as reduced progression of T-ALL proliferation in a murine leukemia model. Furthermore, IRAK knockdown, along with ABT-737 or vincristine treatment, strikingly increased survival of mice. This study suggests that inhibition of IRAK4 signaling pathway has a therapeutic potential to improve chemotherapeutic efficacy [68]. IRAK1 and IRAK4 are implicated in the progression and pathogenesis of Vogt-Koyanagi-Harada (VKH) disease. High levels of IRAK1 and IRAK4 mRNA were found in VKH patients, as compared to those in normal patients. Marked reduction in CD4(+) T cells was observed in the patients administered with dual IRAK1/4 inhibitors. IRAK1/4 inhibition was also associated with a decreased expression of IFN-γ and IL-17 [69].

## 8. Concluding Remarks

The therapeutic potential of IRAK4 has been justified by a number of successful drug discovery programs, which have provided highly potent IRAK4 blockers. However, most of these inhibitors suffered from poor kinase selectivity and weak whole-blood potency, limiting their use in pre-clinical models. Many leads have been discovered to date, as a result of aggressive HTS and medicinal chemistry programs [37,70]. Kinase drug discovery programs face a fundamental challenge of obtaining desired selectivity over off-target kinases, which causes adverse effects on the host. Moreover, most kinases work in a variety of overlapping signaling cascades, and inhibition of multiple kinases can make the interpretation of results from functional assays and efficacy studies difficult. A kinase selective compound must show two key interactions with the enzyme catalytic site residues: H-bonds with the hinge (IRAK4-Met265) and catalytic lysine (IRAK4-Lys213), and a π-stacking interaction with the gatekeeper (IRAK4-Tyr262). Since the bulky tyrosine gatekeeper is unique to the IRAK family, this site has been explored by many groups, using SAR, to obtain better selectivity and reduce off-target binding. Another key concern in the development of IRAK4 inhibitors is the contribution of IRAK1 to the cellular potency of the compounds, indicating that dual inhibition of IRAK4 and IRAK1 may be necessary to block the production of pro-inflammatory cytokines.
